# Transdiagnostic alterations in white matter microstructure associated with suicidal thoughts and behaviours in the ENIGMA Suicidal Thoughts and Behaviours consortium

**DOI:** 10.1038/s41398-025-03602-1

**Published:** 2025-10-24

**Authors:** Laura S. van Velzen, Lejla Colic, Zuriel Ceja, Maria R. Dauvermann, Luca M. Villa, Hannah S. Savage, Yara J. Toenders, Niousha Dehestani, Alyssa H. Zhu, Adrian I. Campos, Lauren E. Salminen, Martin Alda, Ingrid Agartz, Nina Alexander, Rosa Ayesa-Arriola, Elizabeth D. Ballard, Nerisa Banaj, Carlotta Barkhau, Zeynep Başgöze, Jochen Bauer, Francesco Benedetti, Klaus Berger, Bianca Besteher, Katharina Brosch, Manuel Canal-Rivero, Simon Cervenka, Romain Colle, Colm G. Connolly, Emmanuelle Corruble, Philippe Courtet, Baptiste Couvy-Duchesne, Benedicto Crespo-Facorro, Kathryn R. Cullen, Udo Dannlowski, Jeremy Deverdun, Ana M. Diaz-Zuluaga, Lorielle M. F. Dietze, Jennifer W. Evans, Negar Fani, Kira Flinkenflügel, Naomi P. Friedman, Ian H. Gotlib, Nynke A. Groenewold, Dominik Grotegerd, Tomas Hajek, Alexander S. Hatoum, Marco Hermesdorf, Ian B. Hickie, Yoshiyuki Hirano, Tiffany C. Ho, Yuki Ikemizu, Frank Iorfino, Jonathan C. Ipser, Yuko Isobe, Andrea P. Jackowski, Fabrice Jollant, Tilo Kircher, Melissa Klug, Sheri-Michelle Koopowitz, Anna Kraus, Axel Krug, Emmanuelle Le Bars, Elisabeth J. Leehr, Meng Li, Elizabeth T. C. Lippard, Carlos Lopez-Jaramillo, Ivan I. Maximov, Andrew M. McIntosh, Katie A. McLaughlin, Sean R. McWhinney, Susanne Meinert, Elisa Melloni, Philip B. Mitchell, Benson Mwangi, Igor Nenadić, Stener Nerland, Emilie Olie, Victor Ortiz-García de la Foz, Pedro M. Pan, Fabricio Pereira, Fabrizio Piras, Federica Piras, Sara Poletti, Andrew E. Reineberg, Gloria Roberts, Rafael Romero-García, Matthew D. Sacchet, Giovanni A. Salum, Anca-Larisa Sandu, Carl M. Sellgren, Eiji Shimizu, Harry R. Smolker, Jair C. Soares, J. Douglas Steele, Frederike Stein, Dan J. Stein, Benjamin Straube, Lea Teutenberg, Florian Thomas-Odenthal, Paula Usemann, Romain Valabregue, Johanna Valencia-Echeverry, Gerd Wagner, Gordon Waiter, Martin Walter, Heather C. Whalley, Mon-Ju Wu, Tony T. Yang, Carlos A. Zarate, Andre Zugman, Giovana B. Zunta-Soares, Kees van Heeringen, Sanne J. H. van Rooij, Nic van der Wee, Steven van der Werff, Paul M. Thompson, Hilary P. Blumberg, Anne-Laura van Harmelen, Miguel E. Rentería, Neda Jahanshad, ENIGMA Suicidal Thoughts and Behaviours Consortium, Lejla Colic, Zuriel Ceja, Maria R. Dauvermann, Luca M. Villa, Hannah S. Savage, Yara J. Toenders, Niousha Dehestani, Alyssa H. Zhu, Adrian I. Campos, Lauren E. Salminen, Martin Alda, Ingrid Agartz, Nina Alexander, Rosa Ayesa-Arriola, Elizabeth D. Ballard, Nerisa Banaj, Carlotta Barkhau, Zeynep Başgöze, Jochen Bauer, Francesco Benedetti, Klaus Berger, Bianca Besteher, Katharina Brosch, Manuel Canal-Rivero, Simon Cervenka, Romain Colle, Colm G. Connolly, Emmanuelle Corruble, Philippe Courtet, Baptiste Couvy-Duchesne, Benedicto Crespo-Facorro, Kathryn R. Cullen, Udo Dannlowski, Jeremy Deverdun, Ana M. Diaz-Zuluaga, Lorielle M. F. Dietze, Jennifer W. Evans, Negar Fani, Kira Flinkenflügel, Naomi P. Friedman, Ian H. Gotlib, Nynke A. Groenewold, Dominik Grotegerd, Tomas Hajek, Alexander S. Hatoum, Marco Hermesdorf, Ian B. Hickie, Yoshiyuki Hirano, Tiffany C. Ho, Yuki Ikemizu, Frank Iorfino, Jonathan C. Ipser, Yuko Isobe, Andrea P. Jackowski, Fabrice Jollant, Tilo Kircher, Melissa Klug, Sheri-Michelle Koopowitz, Anna Kraus, Axel Krug, Elisabeth J. Leehr, Meng Li, Elizabeth T. C. Lippard, Carlos Lopez-Jaramillo, Ivan I. Maximov, Andrew M. McIntosh, Katie A. McLaughlin, Sean R. McWhinney, Susanne Meinert, Elisa Melloni, Philip B. Mitchell, Benson Mwangi, Igor Nenadić, Stener Nerland, Emilie Olie, Pedro M. Pan, Fabricio Pereira, Fabrizio Piras, Federica Piras, Sara Poletti, Andrew E. Reineberg, Gloria Roberts, Rafael Romero-García, Matthew D. Sacchet, Giovanni A. Salum, Anca-Larisa Sandu, Carl M. Sellgren, Eiji Shimizu, Harry R. Smolker, Jair C. Soares, J. Douglas Steele, Frederike Stein, Dan J. Stein, Benjamin Straube, Lea Teutenberg, Florian Thomas-Odenthal, Paula Usemann, Romain Valabregue, Johanna Valencia-Echeverry, Gerd Wagner, Gordon Waiter, Martin Walter, Heather C. Whalley, Mon-Ju Wu, Tony T. Yang, Carlos A. Zarate, Andre Zugman, Giovana B. Zunta-Soares, Paul M. Thompson, Hilary P. Blumberg, Miguel E. Rentería, Neda Jahanshad, Laura S. van Velzen, Emmanuelle Le Bars, Victor Ortiz-García de la Foz, Kees van Heeringen, Sanne J. H. van Rooij, Nic van der Wee, Steven van der Werff, Anne-Laura van Harmelen, Lianne Schmaal, Lianne Schmaal

**Affiliations:** 1 Orygen, Parkville, VIC Australia; 2 Centre for Youth Mental Health, University of Melbourne, Melbourne, VIC Australia; 3 Department of Psychiatry, Yale University School of Medicine, New Haven, CT USA; 4 Department of Psychiatry and Psychotherapy, Jena University Hospital, Jena, Germany; 5 DZPG (German Center for Mental Health), partner site Halle/Jena/Magdeburg, Halle/Jena/Magdeburg, Germany; 6 QIMR Berghofer Medical Research Institute, Brisbane, QLD Australia; 7 School of Biomedical Sciences, Faculty of Medicine, The University of Queensland, Brisbane, QLD Australia; 8 Institute for Mental Health, School of Psychology, University of Birmingham, Birmingham, UK; 9 Department of Psychiatry, University of Cambridge, Cambridge, UK; 10 Melbourne Neuropsychiatry Centre, Department of Psychiatry, The University of Melbourne, Victoria, Australia; 11 Department of Psychology, Education and Child Studies, Erasmus University Rotterdam, Rotterdam, the Netherlands; 12 School of Psychology, Deakin University, Melbourne, VIC Australia; 13 Imaging Genetics Center, Mark and Mary Stevens Neuroimaging and Informatics Institute, Keck School of Medicine, University of Southern California, Marina del Rey, CA USA; 14 Department of Psychiatry, Dalhousie University, Halifax, NS Canada; 15 Norwegian Centre for Mental Disorders Research (NORMENT), Institute of Clinical Medicine, University of Oslo, Oslo, Norway; 16 Department of Psychiatric Research, Diakonhjemmet Hospital, Oslo, Norway; 17 Centre for Psychiatry Research, Department of Clinical Neuroscience, Karolinska Institutet, & Stockholm Health Care Services, Region Stockholm, Stockholm, Sweden; 18 Department of Psychiatry and Psychotherapy, University of Marburg, Marburg, Germany; 19 Center for Mind, Brain, and Behavior (CMBB), University of Marburg, Marburg, Germany; 20 Department of Psychiatry. Marqués de Valdecilla University Hospital, IDIVAL, School of Medicine, University of Cantabria, Santander, Spain; 21 Experimental Therapeutics and Pathophysiology Branch, National Institute of Mental Health, National Institutes of Health, Bethesda, MD USA; 22 Laboratory of Neuropsychiatry, Clinical Neuroscience and Neurorehabilitation Department, Santa Lucia Foundation IRCCS, Rome, Italy; 23 Institute for Translational Psychiatry, University of Münster, Münster, Germany; 24 Department of Psychiatry and Behavioral Sciences, University of Minnesota Medical School, Minneapolis, MN USA; 25 Clinic for Radiology, University Hospital Münster, Münster, Germany; 26 Division of Neuroscience, Psychiatry and Psychobiology Unit, IRCCS San Raffaele Scientific Institute, Milan, Italy; 27 Institute of Epidemiology and Social Medicine, University of Münster, Münster, Germany; 28 Institute of Behavioral Sciences, Feinstein Institutes for Medical Research, Manhasset, NY USA; 29 Instituto de Biomedicina de Sevilla (IBiS)/HUVR/CSIC/Universidad de Sevilla. CIBERSAM (ISCIII), Seville, Spain; 30 Mental Health Service, Hospital Universitario Virgen del Rocío, Sevilla, Spain; 31 Department of Medical Sciences, Psychiatry, Uppsala University, Uppsala, Sweden; 32 MOODS Team, INSERM 1018, CESP, Université Paris-Saclay, Faculté de Médecine Paris-Saclay, Service Hospitalo-Universitaire de Psychiatrie de Bicêtre, Assistance Publique-Hôpitaux de Paris, Hôpitaux Universitaires Paris-Saclay, Hôpital de Bicêtre, Le Kremlin Bicêtre, F-94275 France; 33 Department of Biomedical Sciences, Florida State University, Tallahassee, FL USA; 34 Department of Emergency Psychiatry and Acute Care, Lapeyronie Hospital, CHU Montpellier, Montpellier, France; 35 IGF, Univ. Montpellier, CNRS, INSERM, Montpellier, France; 36 Institute for Molecular Bioscience, the University of Queensland, St Lucia, QLD Australia; 37 Sorbonne University, Paris Brain Institute - ICM, CNRS, Inria, Inserm, AP-HP, Hôpital de la Pitié Salpêtrière, F-75013 Paris, France; 38 Centro de Investigación Biomédica en Red de Salud Mental, Instituto de Salud Carlos III (CIBERSAM), Madrid, Spain; 39 Institut d’Imagerie Fonctionnelle Humaine, I2FH, Department of Neuroradiology, Gui de Chauliac Hospital and University of Montpellier, Montpellier, France; 40 Department of Neuroradiology, Gui de Chauliac Hospital and University of Montpellier, Montpellier, France; 41 Center for Neurobehavioral Genetics,Semel Institute for Neuroscience and Behavior David Geffen School of Medicine, University of California Los Angeles, Los Angeles, CA USA; 42 Research Group in Psychiatry GIPSI, Department of Psychiatry, Faculty of Medicine, Universidad de Antioquia, Medellin, Colombia; 43 Department of Psychiatry and Behavioral Sciences, Emory University, Atlanta, GA USA; 44 Institute for Behavioral Genetics and Department of Psychology and Neuroscience, University of Colorado Boulder, Boulder, CO USA; 45 Department of Psychology, Stanford University, Stanford, CA 94305 USA; 46 Department of Psychiatry & Neuroscience Institute, University of Cape Town, Cape Town, South Africa; 47 Department of Psychological & Brain Sciences, Washington University in St. Louis, St. Louis, MO USA; 48 The University of Sydney, Sydney, Australia; 49 Research Center for Child Mental Development, Chiba University, Chiba, Japan; 50 United Graduate School of Child Development, Osaka University, Kanazawa University, Hamamatsu University, Chiba University and University of Fukui, Fukui, Japan; 51 Department of Psychology, University of California, Los Angeles, Los Angeles, CA USA; 52 Brain Research Institute, University of California, Los Angeles, Los Angeles, CA USA; 53 Department of Cognitive Behavioral Physiology, Graduate School of Medicine, Chiba University, Chiba, Japan; 54 Østfold University College Department of Education, ICT and Learning, Halden, Norway; 55 Universidade Federal de São Paulo, São Paulo, Brazil; 56 Faculty of Medicine, University Paris-Saclay, Le Kremlin-Bicetre, France; 57 Clinical Department of Psychiatry and Addictology, Paul Brousse Hospital, APHP, Villejuif, France; 58 Department of psychiatry, McGill University, Montreal, Canada; 59 Department of Psychiatry, University Hospital Bonn, Bonn, Germany; 60 Department of Psychiatry and Behavioral Sciences, Dell Medical School, University of Texas at Austin, Austin, TX USA; 61 University of Texas at Austin, Austin, TX USA; 62 Department of Health and Functioning, Western Norway University of Applied Sciences, Bergen, Norway; 63 Division of Psychiatry, Centre for Clinical Brain Sciences, University of Edinburgh, Edinburgh, UK; 64 Ballmer Institute for Children’s Behavioral Health, University of Oregon, Eugene, OR USA; 65 Department of Psychology, Harvard University, Cambridge, MA USA; 66 Institute for Translational Neuroscience, University of Münster, Münster, Germany; 67 Discipline of Psychiatry and Mental Health, School of Clinical Medicine, University of New South Wales, Sydney, Australia; 68 Louis A. Faillace, MD, Department of Psychiatry and Behavioral Sciences, McGovern Medical School, The University of Texas Health Science Center at Houston, Houston, TX USA; 69 UTHealth Houston School of Behavioral Health Sciences, Houston, TX USA; 70 MIPA, Université de Nîmes, Nimes, France; 71 Division for clinical research and innovation, University Hospital Center of Nimes, Nimes, France; 72 Institute for Behavioral Genetics, University of Colorado Boulder, Boulder, CO USA; 73 Meditation Research Program, Department of Psychiatry, Massachusetts General Hospital, Harvard Medical School, Boston, MA USA; 74 Child Mind Institute, New York, NY USA; 75 Universidade Federal do Rio Grande do Sul, Hospital de Clinicas de Porto Alegre - Porto Alegre, Porto Alegre, Brazil; 76 Aberdeen Biomedical Imaging Centre, University of Aberdeen, Aberdeen, UK; 77 Department of Physiology and Pharmacology, Karolinska Institutet, Stockholm, Sweden; 78 Institute for Cognitive Science, University of Colorado Boulder, Boulder, CO USA; 79 Division of Imaging Science and Technology, Medical School, University of Dundee, Dundee, UK; 80 SAMRC Unit on Risk & Resilience in Mental Disorders, Department of Psychiatry & Neuroscience Institute, University of Cape Town, Cape Town, South Africa; 81 Institut du Cerveau et de la Moelle épinière, ICM, Inserm U 1127, CNRS UMR 7225, Sorbonne Université, Paris, France; 82 Centre de Neuro-Imagerie de Recherche, CENIR, ICM, Paris, France; 83 Center for Intervention and Research on adaptive and maladaptive brain Circuits underlying mental health (C-I-R-C), Jena-Magdeburg-Halle, Jena, Germany; 84 Generation Scotland, Centre for Genomic and Experimental Medicine, Institute of Genetics and Molecular Medicine, University of Edinburgh, Edinburgh, UK; 85 Department of Psychiatry and Behavioral Sciences, Division of Child and Adolescent Psychiatry, Weill Institute for Neurosciences, University of California San Francisco, San Francisco, CA USA; 86 National Institute of Mental Health, National Institutes of Health, Bethesda, MD USA; 87 Ghent University, Ghent, Belgium; 88 Department of Psychiatry, Leiden University Medical Center, Leiden, The Netherlands; 89 Leiden Institute for Brain and Cognition. Leiden University Medical Center, Leiden, The Netherlands; 90 Department of Radiology and Biomedical Imaging, Yale University School of Medicine, New Haven, CT USA; 91 Child Study Center, Yale School of Medicine, New Haven, CT USA; 92 Education and Child Studies, Leiden University, Leiden, the Netherlands; 93 Present Address: Regeneron Genetics Center, Tarrytown, NY USA

**Keywords:** Depression, Neuroscience

## Abstract

Previous studies have suggested that alterations in white matter (WM) microstructure are implicated in suicidal thoughts and behaviours (STBs). However, findings of diffusion tensor imaging (DTI) studies have been inconsistent. In this large-scale mega-analysis conducted by the ENIGMA Suicidal Thoughts and Behaviours (ENIGMA-STB) consortium, we examined WM alterations associated with STBs. Data processing was standardised across sites, and resulting WM microstructure measures (fractional anisotropy (FA), axial diffusivity (AD), mean diffusivity and radial diffusivity) for 24 WM tracts and one global measure were pooled across 40 cohorts. We compared these measures among individuals with a psychiatric diagnosis and lifetime history of suicide attempt (*n* = 652; mean age = 35.4 ± 14.7; female = 71.8%), individuals with a psychiatric diagnosis but no STB (i.e., clinical controls; *n* = 1871; mean age = 34 ± 14.8; female = 59.8%), and individuals with no psychiatric diagnosis and no STB (i.e., healthy controls; *n* = 642; mean age = 29.6 ± 13.1; female = 62.9%). We also compared these measures among individuals with recent suicidal ideation (*n* = 714; mean age = 36.3 ± 15.3; female = 66.1%), clinical controls (*n* = 1184; mean age = 36.8 ± 15.6; female = 63.1%), and healthy controls (*n* = 1240; mean age = 31.6 ± 15.5; female = 61.0%). We found subtle but statistically significant effects, such as lower FA associated with a history of suicide attempt, over and above the effect of psychiatric diagnoses. These effects were strongest in the corona radiata, thalamic radiation, fornix/stria terminalis, corpus callosum and superior longitudinal fasciculus. Recent suicidal ideation was associated with higher AD in the cingulum. Effect sizes were small (partial eta-squared < 0.015). This large-scale coordinated mega-analysis revealed subtle regional and global alterations in WM microstructure in individuals with a history of suicide attempt and recent suicidal ideation. Longitudinal studies are needed to confirm whether these alterations are a risk factor for suicidal behaviour or ideation.

## Introduction

Suicide is a worldwide public health concern, with more than 700,000 deaths by suicide occurring worldwide annually [1]. For each death by suicide, it is estimated that there are more than 20 attempts, which has a cascading effect that negatively impacts families, friends, and communities [1]. Despite widespread international efforts to reduce deaths by suicide, the number of deaths by suicide continues to increase in several regions of the world [2]. Risk and protective factors for suicidal thoughts and behaviours (STBs) have been identified [3]. Individuals with neuropsychiatric disorders are at higher risk for STBs [4]. Our current understanding of the underlying pathophysiological mechanisms is limited. Therefore, it is crucial that we increase our knowledge and understanding of these neurobiological mechanisms so that we can better target prevention and intervention efforts for individuals with high suicide risk.

Many prior magnetic resonance imaging (MRI) studies have sought to identify neural correlates of suicidal ideation and suicide attempt (for a review please see [5, 6]). However, identifying robust and reliable patterns of neural alterations associated with STBs has been hampered by methodological heterogeneity across studies. Furthermore, due to the high clinical heterogeneity, associations with brain alterations are likely subtle; hence, large samples are needed to increase statistical power and identify neural correlates of STBs. The ENIGMA Suicidal Thoughts and Behaviours (ENIGMA-STB) consortium was established to address these issues by pooling data across international research groups to identify neural correlates of STBs. In our first study of subcortical and cortical grey matter morphology and STBs in young people between 8 and 25 years of age, we identified a subtle association between lifetime history of suicide attempt and the surface area of the frontal pole, a region in the prefrontal cortex [7], in a well-phenotyped subsample that was enriched for STBs and more severe symptoms of major depressive disorder (MDD) or bipolar disorder (BD). In addition to grey matter morphology, alterations in the microstructure of white matter (WM) tracts connecting brain regions may also contribute to risk for STBs. Identifying these WM alterations could reveal new treatment targets and increase the accuracy of monitoring or treatment response prediction.

Previous neuroimaging studies have used diffusion tensor imaging (DTI) to examine microstructural alterations related to STBs (for a review please see [8]). These studies have focused primarily on fractional anisotropy (FA), which measures the coordinated directionality of water diffusion in WM fibre tracts and may reflect the coherence and myelination of neuronal fibres [9, 10]. Previous DTI studies have focused on WM alterations related to suicide attempt in adult MDD patients [11, 12], adolescents and young adults with MDD and BD [13], adults with BD [14], adults with MDD or BD [15, 16], women with borderline personality disorder [17], adolescents and young people with BD [18], adults with panic disorder [19] and adults with psychotic disorders [20]. A lifetime history of suicide attempt has been associated with lower FA values in various regions and WM tracts, including in the prefrontal cortex (PFC) [11, 13, 14], corpus callosum [15–17], cingulum [13], internal capsule [12, 13], uncinate fasciculus [16, 18], and inferior fronto-occipital fasciculus [16]. However, other studies found higher FA in individuals with a history of suicide attempt [19, 20].

Fewer studies have examined associations between FA measures and suicidal ideation, but have investigated WM correlates of recent suicidal ideation in adults with MDD [21], adults with BD [22] and adolescents or young adults with BD/MDD [23]. Findings from these studies suggest that suicidal ideation is associated with lower FA in the corpus callosum [21, 22], uncinate fasciculus [23], and corona radiata [21].”

Thus, few studies have examined WM microstructural alterations related to suicidal ideation, and findings on suicide attempt are inconsistent across studies. Prior studies are hampered by small sample sizes [5], which decreases the likelihood of identifying true effects, increases the probability of false-negative findings, and may cause inflation of effect sizes [24]. In addition, large samples may be needed to identify subtle associations between STBs and WM microstructure. Finally, most prior studies focused on FA and did not examine other WM diffusivity measures, including axial diffusivity (AD), which is associated with axonal number and organisation; mean diffusivity (MD), which may be an estimate of membrane density; and radial diffusivity (RD), which can provide insights into myelination [25, 26]. Previous studies have also focused only on the presence or absence of suicidal ideation or suicide attempt, and have not examined specific aspects of suicide-related thoughts, such as the severity of suicidal ideation.

To address these limitations, we pooled data from 40 cohorts from the ENIGMA-STB consortium to examine associations between measures of WM microstructure (FA, AD, MD and RD) for 24 WM tracts (and one global measure) and STBs in a large transdiagnostic sample. We examined WM microstructure alterations in people with a psychiatric diagnosis and lifetime history of suicide attempt compared to individuals with a diagnosis but no history of suicide attempt (i.e., clinical controls; CLC) and individuals with no disorder and no history of suicide attempt (healthy controls; HC). In addition, we examined WM alterations in people with recent suicidal ideation (within the last six months) but no history of suicide attempt, compared to CLC and HC.

Based on previous findings concerning suicidal behaviour, we expected that a lifetime history of suicide attempt would be associated with lower FA in WM tracts that connect the frontal lobe with limbic regions or connect different limbic regions, such as the cingulum, corpus callosum, internal capsule, and uncinate fasciculus [12, 13, 15, 18, 23].

In a subsample of participants for whom more in-depth assessment of STBs from the Columbia Suicide Severity Rating Scale (CSSRS) was available, we investigated associations between WM microstructure and the severity of both lifetime and recent suicidal ideation. Finally, in this sample, we were able to examine associations between WM microstructure and suicide attempt, and also distinguish among interrupted, aborted, and actual suicide attempt.

## Patients and methods

### Cohorts

We pooled data from 40 international cohorts from 15 countries (see Supplementary Figure 1) to investigate the association between STBs and WM microstructure in a transdiagnostic sample, including individuals diagnosed with major depressive disorder, obsessive-compulsive disorder, bipolar disorder, post-traumatic stress disorder, psychotic disorders, generalised anxiety disorder, panic disorder, or social anxiety disorder (please see Supplemental Note 1 for an overview of the diagnoses). Demographic characteristics of the different samples are presented in Table 1 and Table 2. The inclusion and exclusion criteria for the different studies are shown in Supplemental Table S1.

**Table 1:** Descriptive statistics for sites included in the lifetime suicide attempt analyses.

Site	Age HC	Age CLC	Age suicide attempt group	% female HC	% female CLC	% female suicide attempt group	Total N HC	Total N CLC	Total N suicide attempt group
BiDirect	–	48.6 (7.3)	49.5 (7.3)	–	56.3	66.9	0	316	124
CHU Montpellier BICS	42.2 (11.7)	45.3 (14.4)	44.2 (13.8)	71.4	86.4	90.0	21	22	20
CHU Montpellier IMPACT	–	38.3 (12.9)	38.6 (9.8)	–	94.4	85.7	0	18	7
CHU Montpellier UF8555	37.3 (7.9)	35.4 (8.4)	37.9 (9.5)	100.0	100.0	100.0	28	47	42
DCHS	30.9 (5.6)	29.7 (6.4)	32.3 (6.0)	100.0	100.0	100.0	22	20	9
MOODS team DEP-ARREST CLIN	35.5 (13.0)	33.2 (10.2)	34.8 (14.3)	67.7	70.8	63.0	31	24	27
ETPB-STB	33.9 (11.3)	34.3 (7.9)	34.8 (11.9)	65.0	50.0	63.6	20	12	11
Fondazione Santa Lucia _ Neuropsychiatry Lab / (FSL_NPL)- OCD sample	39.3 (10.5)	39.9 (11.1)	38.1 (7.6)	35.4	34.9	36.4	82	43	11
FSL_NPL - Schizophrenia sample	–	40.7 (11.7)	43.9 (9.9)	–	32.7	44.4	0	49	18
Ghent	33.6 (13.4)	38.2 (11.7)	34.8 (11.9)	94.1	53.3	69.2	17	15	13
GIPSI BD	–	31.5 (10.3)	34.4 (11.3)	–	25.0	25.0	0	24	8
GIPSI SZ	–	39.7 (11.9)	40.0 (11.4)	–	62.2	68.0	0	45	25
Grady Trauma Project Emory University	39.4 (13.0)	39.3 (12.1)	36.1 (10.9)	97.8	100.0	100.0	46	51	14
Halifax	40.4 (13.7)	42.8 (17.8)	51.0 (16.5)	64.3	41.2	57.1	42	17	7
Houston BD	24.1 (14.7)	29.1 (16.4)	30.2 (11.2)	43.1	48.4	64.3	51	64	14
LTS Colorado site 1	28.7 (1.1)	28.6 (0.9)	28.5 (0.5)	63.6	37.0	76.9	55	92	13
LTS Colorado site 2	28.6 (0.6)	28.7 (0.8)	28.6 (0.5)	73.3	62.5	66.7	30	72	9
McGill University	35.8 (8.0)	44.5 (11.3)	39.4 (10.6)	53.3	50.0	77.8	15	14	9
MR-IMPACT	–	15.0 (1.4)	15.2 (1.2)	–	67.1	91.4	0	70	35
PAFIP	–	29.8 (8.5)	27.3 (7.8)	–	36.4	26.7	0	77	15
San Raffaele Hospital	–	47.7 (10.9)	46.7 (10.2)	–	63.5	74.7	0	288	79
Sydney Bipolar Kids and Siblings	20.9 (3.6)	23.0 (4.8)	24.1 (4.0)	52.2	65.5	75.0	67	84	24
Sydney Brain and Mind Centre	–	19.5 (3.5)	18.1 (2.6)	–	65.1	68.8	0	169	32
UCSF Adolescent MDD	15.4 (1.4)	15.7 (1.3)	16.0 (1.1)	60.0	55.8	71.4	40	43	14
University of MinnesotaAdolescent MDD	16.5 (2.2)	15.5 (1.8)	16.9 (2.1)	72.7	78.9	71.4	11	38	7
University of Texas- Austin -Bipolar Seed Program	20.5 (1.7)	21.0 (2.3)	22.4 (2.0)	68.4	73.3	57.1	19	15	7
University of Washington/ Harvard	12.0 (1.8)	12.4 (2.6)	14.4 (1.8)	46.7	35.5	60.0	45	31	5
Yale School of Medicine	–	19.3 (3.4)	19.0 (3.2)	–	60.4	73.6	0	111	53
**Total**	**29.6 (13.1)**	**34.0** (**14.8)**	**35.4** (**14.7)**	**62.9**	**59.8**	**71.8**	**642**	**1871**	**652**

Presented here are age (mean and standard deviation) and sex for the three groups (*HC* healthy controls, *CLC* clinical controls, group with lifetime suicide attempt) for the different sites included in the analysis on lifetime history of suicide attempt.

**Table 2:** Descriptive statistics for studies included in the recent suicidal ideation analysis.

Site	Age HC	Age CLC	Age suicidal ideation group	% female HC	% female CLC	% female suicidal ideation group	Total N HC	Total N CLC	Total N suicidal ideation group
BiDirect	–	48.7 (7.3)	48.3 (7.0)	–	57.0	65.2	0	405	46
Brazilian High Risk Cohort Porto Alegre	11.0 (2.0)	11.2 (1.9)	11.2 (1.9)	48.1	61.9	80.0	181	21	5
Chiba University	31.2 (8.4)	34.1 (6.3)	32.2 (7.8)	20.0	65.0	61.5	5	20	26
CHU Montpellier BICS	42.2 (11.7)	54.5 (10.7)	41.9 (14.3)	71.4	83.3	87.5	21	6	16
CHU Montpellier IMPACT	–	35.3 (12.7)	43.0 (12.6)	–	90.9	100.0	0	11	7
CHU Montpellier UF8555	38.6 (8.2)	36.3 (7.7)	33.9 (9.9)	100.0	100.0	100.0	10	32	14
MOODS team DEP-ARREST-CLIN	35.5 (13.0)	–	31.9 (10.4)	67.7	–	66.7	31	0	18
EPISCA (Leiden adolescents)	14.6 (1.6)	15.9 (2.2)	15.8 (1.7)	85.0	80.0	86.4	20	10	22
ETPB-STB	34.6 (11.8)	–	34.1 (9.0)	72.2	–	62.5	18	0	8
FSL_NPL- OCD sample	–	35.1 (12.3)	38.7 (7.9)	–	39.6	0.0	0	53	6
FOR2107 Marburg	38.1 (13.4)	35.9 (12.8)	35.0 (12.8)	65.4	81.5	57.9	243	27	38
FOR2107 Muenster	29.5 (11.2)	27.6 (9.8)	36.6 (12.7)	64.9	71.0	63.6	154	31	44
Grady Trauma Project Emory University	38.4 (12.4)	41.3 (11.4)	34.7 (12.7)	97.7	97.5	100.0	44	40	11
Jena-SB	–	39.8 (11.1)	37.6 (13.1)	–	53.5	81.8	0	15	22
KASP	–	26.3 (6.1)	26.1 (7.0)	–	52.2	12.5	0	23	8
McGill University	35.8 (7.9)	47.7 (9.6)	42.1 (12.6)	53.3	50.0	50.0	15	6	8
MR-IMPACT	–	15.1 (1.4)	14.8 (1.4)	–	72.5	62.1	0	40	29
Muenster Neuroimaging Cohort	42.1 (10.9)	38.1 (12.0)	36.1 (12.4)	54.2	56.8	58.2	260	37	98
San Raffaele Hospital	–	48.0 (10.0)	48.3 (10.7)	–	63.5	62.9	0	74	151
Stanford University FAA	30.6 (10.2)	35.7 (6.7)	35.5 (9.9)	100.0	100.0	100.0	18	6	8
STRADL site 1	–	52.5 (11.7)	54.4 (13.8)	–	65.0	71.4	0	20	14
STRADL site 2	–	56.3 (10.2)	54.9 (6.4)	–	85.0	75.0	0	20	12
Sydney Brain and Mind Centre	24.0 (4.3)	20.0 (4.3)	19.7 (4.9)	56.0	59.8	69.8	75	112	43
TIPS-Jena	47.4 (16.1)	46.8 (12.3)	42.7 (13.0)	51.5	56.0	50.0	66	25	16
UCSF Adolescent MDD	15.4 (1.4)	15.7 (1.3)	15.7 (1.4)	60.0	61.1	28.6	40	36	7
University of Minnesota Adolescent MDD	16.1 (1.9)	15.9 (1.9)	15.6 (2.0)	55.0	75.9	83.3	20	29	24
University of Texas- Austin -Bipolar Seed Program	20.5 (1.7)	21.1 (2.2)	20.8 (2.7)	68.4	70.0	80.0	19	10	5
Yale School of Medicine	–	19.2 (3.5)	18.1 (2.5)	–	57.3	75.0	0	75	8
**Total**	**31.6 (15.5)**	**36.8 (15.6)**	**36.3** (**15.3)**	**61.0**	**63.1**	**66.1**	**1240**	**1184**	**714**

Presented here are age (mean and standard deviation) and sex for the three groups. *HC* healthy controls, *CLC* clinical controls, group with recent suicidal ideation) for the different sites included in the analysis on suicidal ideation.

### Ethics approval and consent

All cohorts obtained ethics approval from their local institutional review boards and ethics committees. Participants who were 18 years old and over provided written informed consent; those under the age of 18 years provided written informed assent in addition to written informed consent from a parent/guardian at the local institution. Data acquisition and analysis were performed in accordance with the relevant guidelines and regulations.

### Image processing

Scanner characteristics and acquisition parameters for all cohorts are provided in Supplemental Table S2. Each site performed local preprocessing of diffusion-weighted images, including diffusion tensor fitting. The pre-processed images were then processed using the ENIGMA-DTI protocol, including quality control procedures. This protocol is freely available on the ENIGMA-GitHub webpage (https://github.com/ENIGMA-git#enigma-dti-imaging) and NITRC (http://www.nitrc.org/projects/enigma_dti). Details are provided in Supplemental Note 2. For 24 tracts of interest, FA, AD, MD and RD measures were extracted (please see Supplemental Table S3). We combined regions of interest (ROIs) across hemispheres by calculating the mean of the left and right hemispheres, weighted by the number of voxels, in order to reduce the number of statistical tests. In addition, a global anisotropy or diffusivity measure was created, leading to a total of 25 measures for FA, AD, MD, and RD.

The WM microstructure measures were harmonised across sites using the ComBat algorithm in R [27, 28]. An empirical Bayes approach was used to adjust for variability between scanners while preserving biological variability related to age, sex, and diagnosis. In line with our previous study [7], all DTI measures included in the analyses were ComBat-corrected. Finally, within-site outliers (measures greater than three standard deviations away from the mean of that region) were visually inspected and, if necessary, excluded from the analysis (please see Supplemental Note 2).

### Statistical analysis

The main aim of this study was to examine associations among WM microstructure, lifetime history of suicide attempt, and recent (in the last six months) suicidal ideation in a large transdiagnostic sample. The cohorts included in this multi-study analysis administered different instruments to assess recent suicidal ideation and lifetime history of attempt. We used a similar approach to our previous study [7] to harmonise these measures across studies (see Supplemental Table S4). Lifetime history of suicide attempt (coded yes/no) was determined using clinical interviews (e.g., the Kiddie Schedule for Affective Disorders and Schizophrenia (K-SADS [29];) or Structured Clinical Interview for DSM-5 (SCID [30];)) or detailed clinical scales on STBs (e.g., the CSSRS [31] or Self-Injurious Thoughts and Behaviours Interview (SITBI [32];)). Recent suicidal ideation (yes/no in the past six months or more recent) was assessed using items from depression severity rating scales (e.g. the Hamilton Depression Rating Scale (HDRS [33];), Beck Depression Inventory (BDI [34, 35];), detailed clinical scales on STBs (e.g., CSSRS or Beck Scale for Suicidal Ideation (SSI [36, 37];) and clinical interviews (e.g. SCID)). Similar to our previous study [7], we conducted separate analyses for suicidal ideation and suicide attempt to optimise the sample size for each analysis, as only 16 out of the 40 cohorts had information on both suicidal ideation and suicide attempt. In total, we included data from 28 cohorts in the analysis on lifetime suicide attempt and from 28 cohorts in the analysis on recent suicidal ideation.

We assessed differences among groups in global and regional anisotropy or diffusivity measures using ANCOVA analyses in R [38], followed by post-hoc two-group comparisons. We included a group variable to compare WM diffusivity measures between individuals with a lifetime history of suicide attempt (*N* = 652; mean age = 35.4 ± 14.7; female = 71.8%), CLC (*N* = 1871; mean age = 34 ± 14.8; female = 59.8%) and HC (*N* = 642; mean age=29.6 ± 13.1; female = 62.9%). A group variable was also included in separate analyses to compare WM diffusivity measures between individuals with recent suicidal ideation (*N* = 714; mean age = 36.3 ± 15.3; female = 66.1%), CLC (*N* = 1184; mean age = 36.8 ± 15.6; female = 63.1%) and HC (*N* = 1240; mean age = 31.6 ± 15.5; female = 61.0%). Consistent with previous ENIGMA-DTI analyses [39], age, sex, and their linear and non-linear interactions (age-by-sex interaction, age^2^ and age^2^-by-sex interaction) were included as covariates. Effect sizes were reported by the partial eta-squared measure. All p-values were corrected for multiple testing (for the 25 measures per anisotropic/diffusivity measure) using the Benjamini-Hochberg correction in R, resulting in FDR < 0.05. We interpret differences between the STB group and clinical controls to be related to a history of suicide attempt or recent suicidal ideation (depending on the analysis), while differences between the STB group and the HC group (or between the HC and CLC group) may be related to the presence of a psychiatric disorder.

Finally, in supplementary analyses, we examined interactions between group and type of lifetime psychiatric diagnosis in a subsample of participants, as data on lifetime psychiatric diagnosis were not available for all participants (please see Supplemental note 3 for a description of the methods and results).

#### Analysis in the CSSRS sample

We also examined associations between WM microstructure and more detailed phenotypes of suicide attempt and suicidal ideation in a subsample of 7 cohorts that used the CSSRS to assess STBs. The CSSRS was specifically developed to assess the intensity and severity of suicidal thoughts and suicidal behaviour [31]. C-SSRS has good validity, high sensitivity and specificity for suicide attempts [40].

For analyses of suicidal ideation, we examined how the nominal measures of both recent or lifetime severity of suicidal ideation (coded 0–5; 0: no ideation; 1: passive ideation; 2: non-specific active ideation; 3: active ideation with a method, but no plan or intent; 4: active ideation with intent, but no plan; 5: active ideation with a plan and intent) were associated with WM microstructure. For these analyses, the standardised beta was calculated as an effect size.

In line with our previous study, for analyses of suicide attempt, we compared anisotropy/diffusivity measures between individuals with a lifetime history of any attempt (actual, aborted, or interrupted attempt) and individuals with no lifetime history of any attempt [7]. In addition, we compared these measures between individuals with a lifetime history of an actual (non-interrupted and non-aborted) attempt and those without any lifetime suicide attempt. Finally, these measures were compared between individuals with a history of suicidal ideation (but no history of an actual suicide attempt), and those with a lifetime history of an actual attempt. Cohen’s *d* metric was calculated as an effect size for these analyses.

Similar to the main analyses, all analyses in the CSSRS sample included age, sex, age-by-sex, age^2^, and age^2^-by-sex as covariates, and all resulting p-values were corrected for multiple testing (for the 25 measures per anisotropic/diffusivity measure) using the Benjamini-Hochberg correction in R to lead to FDR < 0.05.

## Results

### Lifetime suicide attempt

Results show significant differences in regional AD, FA, MD, RD and differences in global FA and RD between healthy controls (*N* = 642), clinical controls (*N* = 1871) and individuals with a lifetime history of suicide (*N* = 652; please see Supplemental tables S5–S8). Findings from post-hoc tests are presented below.

#### Individuals with a lifetime history of suicide attempt versus HC

Regional AD was higher in the uncinate fasciculus (UNC) in the attempter group compared to HC (*p* = 0.033; Supplemental table S5). In addition, global FA and regional FA in the body of the corpus callosum (BCC), corpus callosum (CC), cingulate gyrus of the cingulum bundle (CGC), corona radiata (CR), external capsule (EC), fornix/stria terminalis (FXST), genu of the corpus callosum (GCC), posterior corona radiata (PCR), posterior thalamic radiation (PTR), splenium of the corpus callosum (SCC), superior longitudinal fasciculus (SLF) and UNC were lower in the attempter group compared to HC (*p* range = 0.001–0.018 ; Supplemental table S6). MD was higher in the BCC, CC, SCC and UNC in the attempter group compared to the HC (*p* = 0.001–0.029*;* Supplemental table S7). Finally, RD was higher in the BCC, CC, CR, FXST, PCR, SCC, SLF and UNC in the attempter group compared to HC (*p* range = 0.006–0.030; Supplemental table S8).

#### Individuals with a lifetime history of suicide attempt versus CLC

Regional FA in the CR, FXST, PCR, PTR, SCC and SLF were lower in the attempter group compared to CLC (*p* range = 0.009–0.024; Supplemental table S6). In addition, regional MD in the UNC was higher in the attempter group compared to CLC (*p* = 0.009*;* Supplemental table S7). Finally, regional RD in the CR, PCR, SLF and UNC was higher in the attempter group compared to CLC (*p* range = 0.018–0.040; Supplemental table S8).

#### CLC versus HC

Finally, lower FA in the BCC, CC, GCC and UNC was lower in the CLC compared to HC (*p* range = 0.016–0.038; Supplemental table S6).

### Recent suicidal ideation

Findings reveal significant differences in regional AD, FA, MD, RD and differences in global AD, FA and RD between healthy controls (*N* = 1240), clinical controls (*N* = 1184) and individuals with recent suicidal ideation (*N* = 714; please see Supplemental tables S9–S12). Findings from post-hoc tests are presented below.

#### Individuals with recent suicidal ideation versus HC

Global FA and regional FA in the anterior corona radiata (ACR), CR, GCC, PCR and superior fronto-occipital fasciculus (SFO) was lower in the ideation group compared to HC (*p* range = 0.001–0.045; Supplemental table S10). Higher regional MD in the SCR and UNC was observed in the ideation group compared to HC (*p* range = 0.012–0.026; Supplemental table S11). Finally, global RD and regional RD in the CR, PTR, SCR and UNC were higher in the ideation group compared to HC (*p* range = 0.005–0.045; Supplemental table S12).

#### Individuals with recent suicidal ideation versus CLC

Global AD and regional AD in the CGC were higher in the ideation group compared to CLC (*p* range = 0.020–0.026; Supplemental table S9).

#### CLC versus HC

Global AD and regional AD in the ACR, CGC, GCC and SLF were lower in the CLC compared to HC (*p* range = 0.001–0.022; Supplemental table S9). Global FA and regional FA in the CC and GCC were lower in the CLC compared to HC (*p* range = 0.005–0.015; Supplemental table S10). Finally, regional MD in the UNC was higher in the CLC compared to HC (*p* = 0.019; Supplemental table S11).

### Deeper phenotyping in the C-SSRS sample

There were no significant associations of regional FA, AD, MD, RD, with severity of lifetime suicidal ideation (*N* = 299; Supplemental tables S13–16) or severity of recent suicidal ideation (*N* = 338; Supplemental tables S17–20). In addition, there were no significant differences in WM microstructure between people with a lifetime history of an actual suicide attempt (*N* = 134) and those without a history of any suicide attempt (no interrupted, aborted, or actual suicide attempt; *N* = 244) (Supplemental tables 21–24). Moreover, there were no significant differences between people with an actual suicide attempt (*N* = 134) and those with lifetime suicidal ideation, but without a history of an actual suicide attempt (*N* = 122; Supplemental tables 25–28). Finally, there were no differences in FA or diffusivity measures between people with a lifetime history of an interrupted, aborted, or actual suicide attempt (*N* = 177) compared to individuals without a lifetime history of any subtype of suicide attempt (*N* = 244; Supplemental tables S29–32).

## Discussion

In this large-scale multi-cohort transdiagnostic analysis, we examined associations between global and regional measures of WM microstructure and STBs in a pooled sample from 40 international cohorts. A lifetime history of suicide attempt was associated with subtle differences in regional FA and diffusivity (higher regional MD and RD), and recent suicidal ideation was associated with higher global AD and regional AD.

Individuals with a lifetime history of suicide attempts had lower regional FA than did clinical controls. This analysis was undertaken to ascertain that the observed differences are not merely attributable to microstructural variances associated with psychiatric disorders, but rather, are significantly influenced by lifetime suicide attempt. The WM tracts that showed this difference between individuals with a lifetime history of suicide attempt and CLC included the *corona radiata* (including the posterior *corona radiata*), part of the fornix (fornix/*stria terminalis*), thalamic radiation (specifically the posterior thalamic radiation), splenium of the corpus callosum, and the superior longitudinal fasciculus. The *corona radiata* and the thalamic radiation connect the cortex with subcortical brain structures, including the brainstem and thalamus. The *corona radiata* is part of the thalamic-cortical circuitry and has been associated with perceptual, motor, emotional and cognitive function, including behavioural regulation, and lower FA in the *corona radiata* has been associated with poorer executive functioning [41–44]. In addition, previous single-cohort studies have reported that lower FA in the corona radiata is associated with suicidal behaviour [16, 45], albeit inconsistently [20]. Moreover, lower FA in the *corona radiata* predicted suicidal behaviour on average two years later [46]. The (posterior) thalamic radiations also connect the thalamus with cortical regions, including the parietal and occipital lobes. These are important for cortical arousal and consciousness but may also be associated with cognitive control [47]. While lower FA in this tract has not been associated with suicidal behaviour before, FA in this tract was lower in individuals with BD [48], who are at higher risk of suicidal behaviour [49]. The splenium of the corpus callosum connects the occipital-parietal and temporal cortex from both hemispheres and has been associated with visuospatial functioning, reading, language processing, and consciousness [50]. Lower FA in the splenium of the corpus callosum has been associated with a higher number of suicide attempts in BD and MDD [15]. The fornix (including the fornix/*stria terminalis*) connects the amygdala to the hypothalamus and plays an important role in threat monitoring, regulation of the hypothalamic-pituitary-adrenal (HPA) axis and behavioural inhibition [51]. In addition, previous investigators have speculated that impaired structural connectivity in this tract may contribute to feelings of anhedonia [52], which may drive suicidality [53]. Finally, the superior longitudinal fasciculus is an association tract that connects the parietal and temporal cortex with the frontal cortex and may play a role in speech, spatial awareness, processing speed, and attention [54]. FA in this region has inconsistently been associated with suicidality (see [20, 55] and has been consistently linked to brooding and rumination [56], which are risk factors for suicidal behaviour [57].

In this large sample, we were unable to replicate previous findings of lower FA in other WM tracts, including the cingulum [13], internal capsule [12, 13], uncinate fasciculus [16, 18], and inferior fronto-occipital fasciculus [16]. This lack of replication may be related to the small sample size of these previous studies, related to sample characteristics, or be specific to certain psychiatric disorders.

We also found other differences in diffusivity between individuals with a history of suicide attempt and CLC: higher regional RD in the corona radiata, SLF and higher regional MD and RD in the uncinate fasciculus in the suicide attempter group compared to the CLC. The uncinate fasciculus is a long-range association pathway that connects the prefrontal cortex to the anterior temporal lobe and amygdala and plays an important role in emotion regulation, memory retrieval, decision making and reward sensitivity (please see [58] for a review). We previously observed lower frontal pole surface area in young people with a history of suicide attempt [7]. Together with our current findings in the uncinate fasciculus, we could speculate that these alterations in WM microstructure might lead to reduced connectivity and lead to atrophy in prefrontal regions such as the frontal pole, or that reduced surface area in the frontal pole is associated with reduced demand for connectivity, leading to changes in WM microstructure in the UF tract.

Our findings provide evidence consistent with previous ENIGMA reports about differences in WM microstructure in major depressive disorder [39], bipolar disorder [59] and schizophrenia [60] with DTI data, where associations with global and regional FA were reported. The MDD study included 1305 individuals diagnosed with MDD and 1602 HC; the results showed lower global FA and regional FA in 16 out of 24 WM tracts and higher RD in adult individuals with recurrent MDD; the largest FA differences were observed in the corpus callosum and *corona radiata* [39]. On the other hand, the schizophrenia study included 1963 individuals diagnosed with schizophrenia and 2359 HC, and the results showed lower global FA and regional FA in 20 out of 24 ROIs; the larger differences were reported in anterior *corona radiata* and corpus callosum [60]. Finally, the study on bipolar disorder included 1482 individuals diagnosed with BD and 1551 HC and showed lower global FA and regional FA in 29 out of 43 tracts in patients, most prominently in the corpus callosum and cingulum [59].

In a subsample of 7 cohorts, which had more deeply phenotyped suicidal thoughts and behaviours using the CSSRS, we were able to examine the associations with more detailed phenotypes of suicide attempt and suicidal ideation. In our previous study [7] on grey matter morphology and STB in young people, lower surface area of the frontal pole was associated with a history of actual (non-interrupted or non-aborted) suicide attempt in a subsample of studies that had used the CSSRS to assess suicidal behaviour (4 out of 7 cohort overlap, 53% total sample overlap with the CSSRS sample in the current study). In this study, we did not find any associations between WM anisotropy or diffusivity measures and severity of suicidal ideation, nor did we find differences in these measures related to suicide attempt in general (actual, interrupted or aborted suicide attempt), or actual suicide attempt specifically (non-interrupted or non-aborted suicide attempts). It is possible that this sample was not large enough to identify subtle associations between suicide attempt and WM microstructure.

This study increases our understanding of the mechanisms underlying suicidal behaviour by confirming findings from previous smaller studies that lower FA is implicated in suicidal behaviour and showing which tracts are involved. However, the effect sizes for significant group differences observed in this study were small (all d < 0.25), especially for differences between the suicidal ideation/suicide attempter groups and the CLC. High clinical heterogeneity may mask larger effects in individual persons, which we were not able to detect with the group average approach used in the current study. Nonetheless, the currently observed differences are too subtle to be clinically useful in terms of prediction of risk for suicidal behaviour at the individual level. In addition, it is unclear whether alterations in WM microstructure represent a risk factor for suicidal behaviour or are a consequence of a previous suicide attempt. Previous longitudinal studies have shown that lower regional FA at baseline predicts suicidal behaviour at follow-up in individuals with mood disorders [13, 46]. Further, FA has been found to be highly heritable [61], suggesting that alterations in WM microstructure may indeed represent a risk factor for suicidal behaviour. However, cellular brain damage following hypoxia/anoxia during a suicide attempt may also cause WM damage and affect WM microstructure [62].

A strength of this study is the large sample size, which allowed the examination of more detailed phenotypes in subsamples. A second strength is the use of harmonised protocols for diffusion image processing and quality control. Third, we were able to include many samples and show that the results that we find are robust across many independent samples, which also differ in terms of geographical location, cultural background, demographic and clinical characteristics. However, we should also acknowledge several limitations of this study, including heterogeneity across samples in how STBs were assessed. We have tried to minimise this effect by using a detailed process to harmonise these measures across studies, by selecting only dichotomous outcomes that showed moderate to high concurrent validity across measures (Campos et al., 2023) and if sites had multiple measures available within a sample, by only selecting measures that showed high concurrent validity with other measures (Campos et al., 2023). While we chose to only examine lifetime history of suicide attempt and presence of suicidal ideation for this reason, future studies should focus on more detailed measures of STBs, including for instance the number of suicide attempts and lethality of attempts. In addition to differences in clinical assessments, other sources of heterogeneity—such as BMI, medication class, and time since suicide attempt—may have introduced unmeasured variability and reduced our ability to detect subtle white matter effects. While we used harmonization techniques to address between-site differences, the disproportionate contribution of larger cohorts and variation in acquisition protocols, clinical measures, and sample characteristics may still have obscured small but meaningful effects. Finally, we could not control for severity of psychiatric symptoms, as this was a transdiagnostic analysis and the different cohorts had used different severity rating scales; therefore, we cannot rule out the possibility that higher symptom severity in the suicide attempter group affected our findings. The scope of this study was limited to cross-sectional findings. However, this presents an opportunity for future research to delve into longitudinal analyses, shedding more light on the causal association between WM alterations and STBs. These future longitudinal analyses would require a large sample size at baseline given the low base rate of suicidal behaviour. Finally, our results may not generalise to individuals with STBs without a psychiatric disorder, which may represent a distinct subgroup with unique risk factors and underlying neurobiological mechanisms.

To conclude, in this large-scale multi-cohort study we found an association between a history of suicide attempt and white matter microstructure, above and beyond the effect of psychiatric diagnosis. Measures of white matter microstructure in several tracts that have been associated with (among others) behavioural regulation/inhibition, executive functioning, and brooding/rumination show a subtle association with suicidal attempt. Future longitudinal studies are needed to examine if altered white matter microstructure may represent a risk factor for suicidal behaviour, for instance by contributing to lower resilience to severely stressful life events.

## Supplementary information

Supplemental material

## Data Availability

The datasets analysed in the current study are not publicly available due to privacy restrictions.

## References

[1] WHO. Suicide worldwide in 2019: global health estimates. 2021. https://apps.who.int/iris/bitstream/handle/10665/341728/9789240026643-eng.pdf?sequence=1.

[2] Martínez-Alés G, Jiang T, Keyes KM, Gradus JL. The recent rise of suicide mortality in the United States. Annu Rev Public Health. 2022;43:99–116. 34705474 10.1146/annurev-publhealth-051920-123206 PMC11107879

[3] Wasserman D, Carli V, Iosue M, Javed A, Herrman H. Suicide prevention in childhood and adolescence: a narrative review of current knowledge on risk and protective factors and effectiveness of interventions. Asia Pac Psychiatry. 2021;13:e12452. 33646646 10.1111/appy.12452

[4] Favril L, Yu R, Uyar A, Sharpe M, Fazel S. Risk factors for suicide in adults: systematic review and meta-analysis of psychological autopsy studies. Evid Based Ment Health. 2022;25:148–55. 36162975 10.1136/ebmental-2022-300549 PMC9685708

[5] Schmaal L, van Harmelen A-L, Chatzi V, Lippard ETC, Toenders YJ, Averill LA, et al. Imaging suicidal thoughts and behaviors: a comprehensive review of 2 decades of neuroimaging studies. Mol Psychiatry. 2020;25:408–27. 31787757 10.1038/s41380-019-0587-x PMC6974434

[6] Auerbach RP, Pagliaccio D, Allison GO, Alqueza KL, Alonso MF. Neural correlates associated with suicide and nonsuicidal self-injury in youth. Biol Psychiatry. 2021;89:119–133. 32782140 10.1016/j.biopsych.2020.06.002 PMC7726029

[7] van Velzen LS, Dauvermann MR, Colic L, Villa LM, Savage HS, Toenders YJ, et al. Structural brain alterations associated with suicidal thoughts and behaviors in young people: results from 21 international studies from the ENIGMA Suicidal Thoughts and Behaviours consortium. Mol Psychiatry. 2022;27:4550–60. 36071108 10.1038/s41380-022-01734-0 PMC9734039

[8] Zanghì E, Corallo F, Lo Buono V. Diffusion tensor imaging studies on subjects with suicidal thoughts and behaviors: a descriptive literature review. Brain Behav. 2022;12:e2711. 35943210 10.1002/brb3.2711 PMC9480894

[9] Podwalski P, Szczygieł K, Tyburski E, Sagan L, Misiak B, Samochowiec J. Magnetic resonance diffusion tensor imaging in psychiatry: a narrative review of its potential role in diagnosis. Pharmacol Rep. 2021;73:43–56. 33125677 10.1007/s43440-020-00177-0 PMC7862529

[10] Jones DK, Knösche TR, Turner R. White matter integrity, fiber count, and other fallacies: the do’s and don’ts of diffusion MRI. Neuroimage. 2013;73:239–54. 22846632 10.1016/j.neuroimage.2012.06.081

[11] Olvet DM, Peruzzo D, Thapa-Chhetry B, Sublette ME, Sullivan GM, Oquendo MA, et al. A diffusion tensor imaging study of suicide attempters. J Psychiatr Res. 2014;51:60–7. 24462041 10.1016/j.jpsychires.2014.01.002 PMC4060601

[12] Jia Z, Huang X, Wu Q, Zhang T, Lui S, Zhang J, et al. High-field magnetic resonance imaging of suicidality in patients with major depressive disorder. Am J Psychiatry. 2010;167:1381–90. 20843871 10.1176/appi.ajp.2010.09101513

[13] Lippard ETC, Cox Lippard ET, Johnston JAY, Spencer L, Quatrano S, Fan S, et al. Preliminary examination of gray and white matter structure and longitudinal structural changes in frontal systems associated with future suicide attempts in adolescents and young adults with mood disorders. Journal of Affective Disorders. 2019;245:1139–48. 30699858 10.1016/j.jad.2018.11.097 PMC6487887

[14] Mahon K, Burdick KE, Wu J, Ardekani BA, Szeszko PR. Relationship between suicidality and impulsivity in bipolar I disorder: a diffusion tensor imaging study. Bipolar Disord. 2012;14:80–9. 22329475 10.1111/j.1399-5618.2012.00984.x PMC3319758

[15] Cyprien F, de Champfleur NM, Deverdun J, Olié E, Le Bars E, Bonafé A, et al. Corpus callosum integrity is affected by mood disorders and also by the suicide attempt history: A diffusion tensor imaging study. J Affect Disord. 2016;206:115–24. 27472413 10.1016/j.jad.2016.07.026

[16] Wei S, Womer FY, Edmiston EK, Zhang R, Jiang X, Wu F, et al. Structural alterations associated with suicide attempts in major depressive disorder and bipolar disorder: A diffusion tensor imaging study. Prog Neuropsychopharmacol Biol Psychiatry. 2020;98:109827. 31778758 10.1016/j.pnpbp.2019.109827

[17] Lischke A, Domin M, Freyberger HJ, Grabe HJ, Mentel R, Bernheim D, et al. Structural Alterations in the Corpus Callosum Are Associated with Suicidal Behavior in Women with Borderline Personality Disorder. Front Hum Neurosci. 2017;11:196. 28484382 10.3389/fnhum.2017.00196 PMC5401902

[18] Johnston JAY, Wang F, Liu J, Blond BN, Wallace A, Liu J, et al. Multimodal neuroimaging of frontolimbic structure and function associated with suicide attempts in adolescents and young adults with bipolar disorder. Am J Psychiatry. 2017;174:667–75. 28135845 10.1176/appi.ajp.2016.15050652 PMC5939580

[19] Kim B, Oh J, Kim M-K, Lee S, Tae WS, Kim CM, et al. White matter alterations are associated with suicide attempt in patients with panic disorder. J Affect Disord. 2015;175:139–46. 25617685 10.1016/j.jad.2015.01.001

[20] Lee S-J, Kim B, Oh D, Kim M-K, Kim K-H, Bang SY, et al. White matter alterations associated with suicide in patients with schizophrenia or schizophreniform disorder. Psychiatry Res Neuroimaging. 2016;248:23–9. 26774424 10.1016/j.pscychresns.2016.01.011

[21] Reis JV, Vieira R, Portugal-Nunes C, Coelho A, Magalhães R, Moreira P, et al. Suicidal Ideation Is Associated With Reduced Functional Connectivity and White Matter Integrity in Drug-Naïve Patients With Major Depression. Front Psychiatry. 2022;13:838111. 35386522 10.3389/fpsyt.2022.838111 PMC8978893

[22] Zhang R, Jiang X, Chang M, Wei S, Tang Y, Wang F. White matter abnormalities of corpus callosum in patients with bipolar disorder and suicidal ideation. Ann Gen Psychiatry. 2019;18:20. 31528196 10.1186/s12991-019-0243-5 PMC6737682

[23] Fan S, Lippard ETC, Sankar A, Wallace A, Johnston JAY, Wang F, et al. Gray and white matter differences in adolescents and young adults with prior suicide attempts across bipolar and major depressive disorders. J Affect Disord. 2019;245:1089–97. 30699851 10.1016/j.jad.2018.11.095 PMC6903411

[24] Button KS, Ioannidis JPA, Mokrysz C, Nosek BA, Flint J, Robinson ESJ, et al. Power failure: why small sample size undermines the reliability of neuroscience. Nat Rev Neurosci. 2013;14:365–76. 23571845 10.1038/nrn3475

[25] Winklewski PJ, Sabisz A, Naumczyk P, Jodzio K, Szurowska E, Szarmach A. Understanding the physiopathology behind axial and radial diffusivity changes—What Do We Know? Front Neurol. 2018;9:92. 29535676 10.3389/fneur.2018.00092 PMC5835085

[26] Tae WS, Ham BJ, Pyun SB, Kang SH, Kim BJ. Current Clinical Applications of Diffusion-Tensor Imaging in Neurological Disorders. J Clin Neurol. 2018;14:129–40. 29504292 10.3988/jcn.2018.14.2.129 PMC5897194

[27] Fortin J-P, Cullen N, Sheline YI, Taylor WD, Aselcioglu I, Cook PA, et al. Harmonization of cortical thickness measurements across scanners and sites. Neuroimage. 2018;167:104–20. 29155184 10.1016/j.neuroimage.2017.11.024 PMC5845848

[28] Radua J, Vieta E, Shinohara R, Kochunov P, Quidé Y, Green MJ, et al. Increased power by harmonizing structural MRI site differences with the ComBat batch adjustment method in ENIGMA. Neuroimage. 2020;218:116956. 32470572 10.1016/j.neuroimage.2020.116956 PMC7524039

[29] Kaufman J, Birmaher B, Brent D, Rao U, Flynn C, Moreci P, et al. Schedule for affective disorders and schizophrenia for school-age children-present and lifetime version (K-SADS-PL): Initial reliability and validity data. J Am Acad Child Adolesc Psychiatry. 1997;36:980–88. 9204677 10.1097/00004583-199707000-00021

[30] First MB. Structured clinical interview for DSM-IV Axis I disorders SCID-I: Clinician version, scoresheet. American Psychiatric Press, 1997.

[31] Posner K, Brent D, Lucas C, Gould M, Stanley B, Brown G, et al. Columbia-suicide severity rating scale (C-SSRS). New York, NY: Columbia University Medical Center 2008. https://depts.washington.edu/ebpa/sites/default/files/C-SSRS-LifetimeRecent-Clinical.pdf.

[32] Nock MK, Holmberg EB, Photos VI, Michel BD. Self-Injurious Thoughts and Behaviors Interview: development, reliability, and validity in an adolescent sample. Psychol Assess. 2007;19:309–17. 17845122 10.1037/1040-3590.19.3.309

[33] Hamilton M. A rating scale for depression. J Neurol Neurosurg Psychiatry. 1960;23:56–62. 14399272 10.1136/jnnp.23.1.56 PMC495331

[34] Beck AT, Ward C, Mendelson M, Mock J, Erbaugh J. Beck depression inventory (BDI). Arch Gen Psychiatry. 1961;4:561–71. 13688369 10.1001/archpsyc.1961.01710120031004

[35] Beck AT, Steer RA, Brown GK. Beck depression inventory-II. San Antonio. 1996;78:490–98.

[36] Beck AT, Kovacs M, Weissman A. Assessment of suicidal intention: the Scale for Suicide Ideation. J Consult Clin Psychol. 1979;47:343–52. 469082 10.1037//0022-006x.47.2.343

[37] Beck AT, Steer RA, Ranieri WF. Scale for suicide ideation: psychometric properties of a self-report version. J Clin Psychol. 1988;44:499–505. 3170753 10.1002/1097-4679(198807)44:4<499::aid-jclp2270440404>3.0.co;2-6

[38] R core team. R: A language and environment for statistical computing. R Foundation for Statistical Computing, Vienna, Austria 2021. https://www.R-project.org/.

[39] van Velzen LS, Kelly S, Isaev D, Aleman A, Aftanas LI, Bauer J, et al. White matter disturbances in major depressive disorder: a coordinated analysis across 20 international cohorts in the ENIGMA MDD working group. Mol Psychiatry. 2020;25:1511–25. 31471575 10.1038/s41380-019-0477-2 PMC7055351

[40] Posner K, Brown GK, Stanley B, Brent DA, Yershova KV, Oquendo MA, et al. The Columbia-Suicide Severity Rating Scale: initial validity and internal consistency findings from three multisite studies with adolescents and adults. Am J Psychiatry. 2011;168:1266–77. 22193671 10.1176/appi.ajp.2011.10111704 PMC3893686

[41] Jenkins LM, Barba A, Campbell M, Lamar M, Shankman SA, Leow AD, et al. Shared white matter alterations across emotional disorders: a voxel-based meta-analysis of fractional anisotropy. Neuroimage Clin. 2016;12:1022–34. 27995068 10.1016/j.nicl.2016.09.001 PMC5153602

[42] Chen Z, Cui L, Li M, Jiang L, Deng W, Ma X, et al. Voxel based morphometric and diffusion tensor imaging analysis in male bipolar patients with first-episode mania. Prog Neuropsychopharmacol Biol Psychiatry. 2012;36:231–38. 22119745 10.1016/j.pnpbp.2011.11.002

[43] Leunissen I, Coxon JP, Caeyenberghs K, Michiels K, Sunaert S, Swinnen SP. Task switching in traumatic brain injury relates to cortico-subcortical integrity. Hum Brain Mapp. 2014;35:2459–69. 23913872 10.1002/hbm.22341 PMC6869801

[44] Wallace EJ, Mathias JL, Ward L. The relationship between diffusion tensor imaging findings and cognitive outcomes following adult traumatic brain injury: A meta-analysis. Neurosci Biobehav Rev. 2018;92:93–103. 29803527 10.1016/j.neubiorev.2018.05.023

[45] Jiang X, Guo Y, Jia L, Zhu Y, Sun Q, Kong L, et al. Altered levels of plasma inflammatory cytokines and white matter integrity in bipolar disorder patients with suicide attempts. Front Psychiatry. 2022;13:861881. 35463510 10.3389/fpsyt.2022.861881 PMC9021603

[46] Colic L, Villa LM, Dauvermann MR, van Velzen LS, Sankar A, Goldman DA, et al. Brain grey and white matter structural associations with future suicidal ideation and behaviors in adolescent and young adult females with mood disorders. JCPP Adv 2022;2. 10.1002/jcv2.12118. 10.1002/jcv2.12118 PMC9937714 36817186

[47] Chaddock-Heyman L, Erickson KI, Voss MW, Powers JP, Knecht AM, Pontifex MB, et al. White matter microstructure is associated with cognitive control in children. Biol Psychol. 2013;94:109–15. 23714226 10.1016/j.biopsycho.2013.05.008 PMC3742734

[48] Hu R, Stavish C, Leibenluft E, Linke JO. White Matter Microstructure in Individuals With and At Risk for Bipolar Disorder: Evidence for an Endophenotype From a Voxel-Based Meta-analysis. Biol Psychiatry Cogn Neurosci Neuroimaging. 2020;5:1104–13. 32839153 10.1016/j.bpsc.2020.06.007 PMC11102922

[49] Dome P, Rihmer Z, Gonda X Suicide risk in bipolar disorder: a brief review. *Medicina* 2019;55. 10.3390/medicina55080403. 10.3390/medicina55080403 PMC6723289 31344941

[50] Blaauw J, Meiners LC. The splenium of the corpus callosum: embryology, anatomy, function and imaging with pathophysiological hypothesis. Neuroradiology. 2020;62:563–85. 32062761 10.1007/s00234-019-02357-z PMC7186255

[51] Clauss J. Extending the neurocircuitry of behavioural inhibition: a role for the bed nucleus of the stria terminalis in risk for anxiety disorders. Gen Psychiatr. 2019;32:e100137. 31922088 10.1136/gpsych-2019-100137 PMC6937153

[52] Harnett NG, Stevens JS, van, Rooij SJH, Ely TD, Michopoulos V, Hudak L, et al. Multimodal structural neuroimaging markers of risk and recovery from posttrauma anhedonia: A prospective investigation. Depress Anxiety. 2021;38:79–88. 33169525 10.1002/da.23104 PMC7785637

[53] Bonanni L, Gualtieri F, Lester D, Falcone G, Nardella A, Fiorillo A, et al. Can anhedonia be considered a suicide risk factor? a review of the literature. Medicina. 2019;55:458. 31405085 10.3390/medicina55080458 PMC6723513

[54] Janelle F, Iorio-Morin C, D’amour S, Fortin D. Superior longitudinal fasciculus: a review of the anatomical descriptions with functional correlates. Front Neurol. 2022;13:794618. 35572948 10.3389/fneur.2022.794618 PMC9093186

[55] Davey DK, Jurick SM, Crocker LD, Hoffman SN, Sanderson-Cimino M, Tate DF, et al. White matter integrity, suicidal ideation, and cognitive dysfunction in combat-exposed Iraq and Afghanistan Veterans. Psychiatry Res Neuroimaging. 2021;317:111389. 34563989 10.1016/j.pscychresns.2021.111389

[56] Pisner DA, Shumake J, Beevers CG, Schnyer DM. The superior longitudinal fasciculus and its functional triple-network mechanisms in brooding. Neuroimage Clin. 2019;24:101935. 31352219 10.1016/j.nicl.2019.101935 PMC6664225

[57] Horwitz AG, Czyz EK, Berona J, King CA. Rumination, brooding, and reflection: prospective associations with suicide ideation and suicide attempts. Suicide Life Threat Behav. 2019;49:1085–93. 30099778 10.1111/sltb.12507 PMC6726493

[58] Olson IR, Von Der Heide RJ, Alm KH, Vyas G. Development of the uncinate fasciculus: implications for theory and developmental disorders. Dev Cogn Neurosci. 2015;14:50–61. 26143154 10.1016/j.dcn.2015.06.003 PMC4795006

[59] Favre P, Pauling M, Stout J, Hozer F, Sarrazin S, Abé C, et al. Widespread white matter microstructural abnormalities in bipolar disorder: evidence from mega- and meta-analyses across 3033 individuals. Neuropsychopharmacology. 2019;44:2285–93. 31434102 10.1038/s41386-019-0485-6 PMC6898371

[60] Kelly S, Jahanshad N, Zalesky A, Kochunov P, Agartz I, Alloza C, et al. Widespread white matter microstructural differences in schizophrenia across 4322 individuals: results from the ENIGMA Schizophrenia DTI Working Group. Mol Psychiatry. 2018;23:1261–9. 29038599 10.1038/mp.2017.170 PMC5984078

[61] Kochunov P, Jahanshad N, Marcus D, Winkler A, Sprooten E, Nichols TE, et al. Heritability of fractional anisotropy in human white matter: a comparison of Human Connectome Project and ENIGMA-DTI data. Neuroimage. 2015;111:300–11. 25747917 10.1016/j.neuroimage.2015.02.050 PMC4387079

[62] DiPoce J, Guelfguat M, DiPoce J. Radiologic findings in cases of attempted suicide and other self-injurious behavior. Radiographics. 2012;32:2005–24. 23150854 10.1148/rg.327125035

